# Cavitation Erosion Behavior of Unalloyed Carbon Steels and Morphology of the Eroded Microstructures

**DOI:** 10.3390/ma19214658

**Published:** 2026-11-01

**Authors:** Ilare Bordeasu, Dorin Bordeasu, Lavinia-Madalina Micu

**Affiliations:** 1 Department of Mechanical Machines, Equipment and Transports, Politehnica University of Timisoara, Bulevardul Mihai Viteazul nr.1, 300222 Timisoara, Romania; ilare.bordeasu@upt.ro; 2 Department of Automation and Applied Informatics, Politehnica University of Timisoara, Vasile Parvan, No. 2, 300223 Timisoara, Romania; 3 Department of Agricultural Technologies-Department I, King Mihai I University of Life Sciences, 119 Calea Aradului, 300645 Timisoara, Romania

**Keywords:** carbon steel, cavitation erosion, cavitation erosion resistance, mass loss, erosion cavities, hardness, microstructure, ferrite, pearlite

## Abstract

Research on the cavitation erosion behavior of carbon steels remains relatively limited, primarily because these materials are conventionally associated with structural applications involving moderate mechanical loading. Nevertheless, a detailed understanding of the response of this class of steels to the impact of shock waves and microjets generated by the implosion of cavitation bubbles is important for engineering applications. This is particularly relevant because components manufactured from carbon steels may operate under cavitating flow conditions characterized by different levels of erosive intensity. Typical examples include hydraulic-machine rotor hubs, valve bodies, hydraulic spools and seats, stator blades, and guide vanes. In this context, the present study investigates the vibratory cavitation erosion behavior of three hypoeutectoid carbon steels: S235J2 general-purpose structural steel, GS300 cast steel, and C15E quality carbon steel. The tests were carried out on the standard 20 kHz vibrating apparatus. The experimental procedure was carried out according to the laboratory’s custom, in compliance with the requirements of ASTM G32. The analysis of the characteristic cavitation erosion curves and parameters, together with macroscopic and microscopic investigations, reveals the superior cavitation erosion resistance of C15E steel, which is associated with its higher hardness, lower elongation at fracture and alloying elements Ni, Cr and Cu.

## 1. Introduction

The erosive effect of cavitation is widely recognized in the literature as the “cancer of hydraulic machinery” [1,2,3,4]. The phenomenon was first observed, although without receiving particular attention at the time, by Francis Pettit Smith [5], on the wrought-iron propeller of the SS Archimedes, built in England in 1838–1839. The subsequent transition from wrought-iron to cast-steel propellers revealed that the erosive effects of cavitation became increasingly severe as vessel propulsion speeds increased.

Consequently, researchers developed experimental facilities capable of generating controlled cavitation conditions [6,7,8,9,10] and initiated extensive investigations aimed at identifying materials with improved resistance to cavitation erosion, particularly for marine propeller applications [11,12,13,14]. Moreover, inspections of rudder blades and ship sterns during maintenance and repair operations revealed clear evidence of cavitation erosion [11,13]. Since these structural components are commonly manufactured from carbon-steel plates, investigating the cavitation erosion resistance of carbon steels, together with developing methods to mitigate cavitation damage, became necessary.

At the same time, the expansion of hydropower generation and water-supply systems has inevitably increased the occurrence of cavitation erosion along hydraulic flow paths, with the most severe damage generally affecting the blades and runners of hydraulic turbines and the impellers of hydraulic pumps [1,2,15,16,17]. Consequently, dedicated experimental laboratories have been established and extensive research programs have been undertaken to investigate the response of material microstructures to the destructive action of microjets and shock waves generated by the implosion of cavitation bubbles [18,19,20,21,22], as well as to optimize flow conditions in order to mitigate cavitation occurrence and intensity [15,23,24,25,26]. In hydraulic machinery, however, cavitation erosion is not restricted to turbine runners and pump impellers. It can also affect the surfaces of components forming the boundaries of the hydraulic flow domain. These include guide-vane rings and guide vanes, commonly manufactured from unalloyed or low-alloy carbon steels [2,3,4,27,28], as well as valve bodies, stator columns, spiral casings, hubs, and nose cones, which are typically manufactured from cast steel [2,3,4,29,30]. Despite their practical importance, the literature provides relatively limited information on the cavitation erosion behavior of these materials and on the morphology and mechanisms of cavitation degradation. Systematic characterization of their response to cavitation loading is therefore required to establish a more comprehensive knowledge base and to support the development of effective strategies for improving their cavitation erosion resistance, including advanced surface-coating and hardening technologies. Furthermore, cavitation erosion has also been observed in components of positive-displacement pumps, such as gears, as well as in flow- and pressure-control devices used in hydraulic actuation systems. Based on our review of the available literature, relatively few studies, either earlier or recent, have addressed cavitation erosion in such components [12,31,32]. The studies identified are predominantly focused on alloy steels and primarily investigate technologies intended to enhance their cavitation erosion resistance [33,34,35,36]. Our research conducted during the troubleshooting and maintenance of such hydraulic devices has shown that cavitation damage occurs particularly frequently on the closure elements of flow- and pressure-regulation and control devices used in hydraulic actuation systems.

Although research on the cavitation erosion behavior of steels belonging to this category has gained some attention in recent years, as demonstrated by the investigations conducted by Krella et al. [37], which highlight the relationships among erosion morphology, microstructure, chemical composition, and mechanical properties, research interest in these materials remains relatively limited. This lack of attention is difficult to explain, particularly considering the increasing costs of alloyed and stainless steels, as well as non-ferrous alloys such as marine bronzes, and the continuous development of advanced technologies for modifying the properties and microstructure of surfaces exposed to cavitation [38].

Accordingly, the present study investigates the behavior and resistance to vibratory cavitation erosion of three hypoeutectoid unalloyed carbon steels, C15E, S235J2, and GS300, currently used in components operating under low-intensity cavitation erosion conditions. The results demonstrate the superior cavitation erosion resistance of C15E steel, which is associated with its higher hardness, lower elongation at fracture, and chemical composition. The findings provide further insights into the cavitation degradation mechanisms of these steels and establish a basis for future investigations involving advanced surface-engineering technologies aimed at improving their cavitation erosion resistance. Such developments may contribute to extending the application of these steels to components operating in liquid environments subjected to more severe cavitation erosion conditions.

## 2. Researched Materials

The researched materials belong to the class of hypoeutectoid unalloyed carbon steels. Three steels were selected: S235J2 general-purpose structural carbon steel, according to SR EN 10025 [39]; GS300 cast steel, according to SR EN 10293 [40]; and C15E case-hardening quality carbon steel, according to SR EN 10084 [41]. Although these steels belong to different categories of iron-carbon alloys, their selection was based on their current use in components operating under cavitating flow conditions characterized by low to moderate erosion intensity. Their typical applications and relevance to the present investigation are summarized below.

- S235J2 general-purpose structural steel is commonly supplied in the form of plates, wires, pipes, and bars with various cross-sectional geometries and is widely used in structural applications, particularly for components subjected to relatively low mechanical loads and requiring good weldability. Nevertheless, this steel is also used in hydraulic equipment, including guide vanes and guide-vane rings, as well as components of hydraulic turbine stators [1,2,3,4,15,28,29]. Under operating conditions, these components may be exposed not only to abrasive erosion but also to localized, low-intensity erosion generated by cavitation.
- GS300 cast steel is commonly used for components subjected to vibration, such as machine-tool frames, as well as for couplings, pinions, and large gears operating under low to moderate mechanical loads. In hydraulic applications, this steel is widely used for turbine runner hubs and spiral casings of hydraulic turbines and turbopumps, as well as for valve bodies and casings of positive-displacement pumps. These components may operate under cavitating flow conditions characterized by low to moderate cavitation erosion intensity [1,2,3,4,7,12,15,29].
- C15E case-hardening quality carbon steel is commonly subjected to carburizing heat treatment and used for components whose surfaces require sufficiently high hardness to withstand erosive loading, particularly abrasive wear [2,33,42]. In hydraulic equipment, C15E steel is used for components such as the spools of hydraulic control devices, including directional control valves and other valve systems, as well as valve seats. Such components have been reported to exhibit erosion damage associated with cavitation [28,33].

At this stage of the investigation, the S235J2 and C15E specimens used for the cavitation erosion tests were machined from commercially available round bars with a diameter of 20 mm. The GS300 specimens were extracted from a section of the hub of an axial-flow hydraulic turbine that had been rejected and subsequently cut for remelting. The material was provided by Uzina de Construcții Mașini Hidroenergetice S.R.L, Reșița, Romania. Accordingly, all three steels were tested in their semi-finished (as-received) state, without any additional bulk heat treatment or surface treatment. This approach was adopted to identify the microstructural features governing the morphology of cavitation degradation and the resistance of the surface microstructure of these steels to cavitation loading in their semi-finished state.

Table 1 and Table 2 present the chemical compositions and mechanical properties, respectively, of the investigated steels. These properties are particularly relevant to the resistance of the material surface to the extremely high local pressures, reaching values on the order of tens of GPa [25,43,44,45], generated by the microjets and shock waves associated with the implosion of cavitation bubbles. The chemical composition and mechanical properties were determined in the specialized Materials Science and Mechanical Testing laboratories of Politehnica University Timișoara (Romania).

The data presented in Table 1 and Table 2 provide a basis for anticipating the influence of chemical composition and mechanical properties on the cavitation erosion resistance of the investigated steels. This assumption is supported by the studies of Bordeasu et al. [38,42,45] conducted on a wide range of steels used for components exposed to cavitation, as well as on more than 30 types of aluminum alloys. These studies investigated the individual influence of mechanical properties on the resistance of the surface microstructure to cavitation erosive loading and suggested a general tendency for materials exhibiting lower elongation and higher hardness to achieve greater cavitation erosion resistance.

Based on this relationship, the mechanical properties reported in Table 2 suggest that C15E steel should exhibit the most favorable cavitation erosion behavior and, consequently, the highest cavitation erosion resistance among the three steels investigated. This expectation is further supported by the presence of Cr, Ni, and Cu. Although present only in small amounts, these elements may contribute to increasing the resistance of the microstructure to the high local pressures generated by the microjets and shock waves associated with cavitation bubble implosion [2,42].

**Figure 1:**
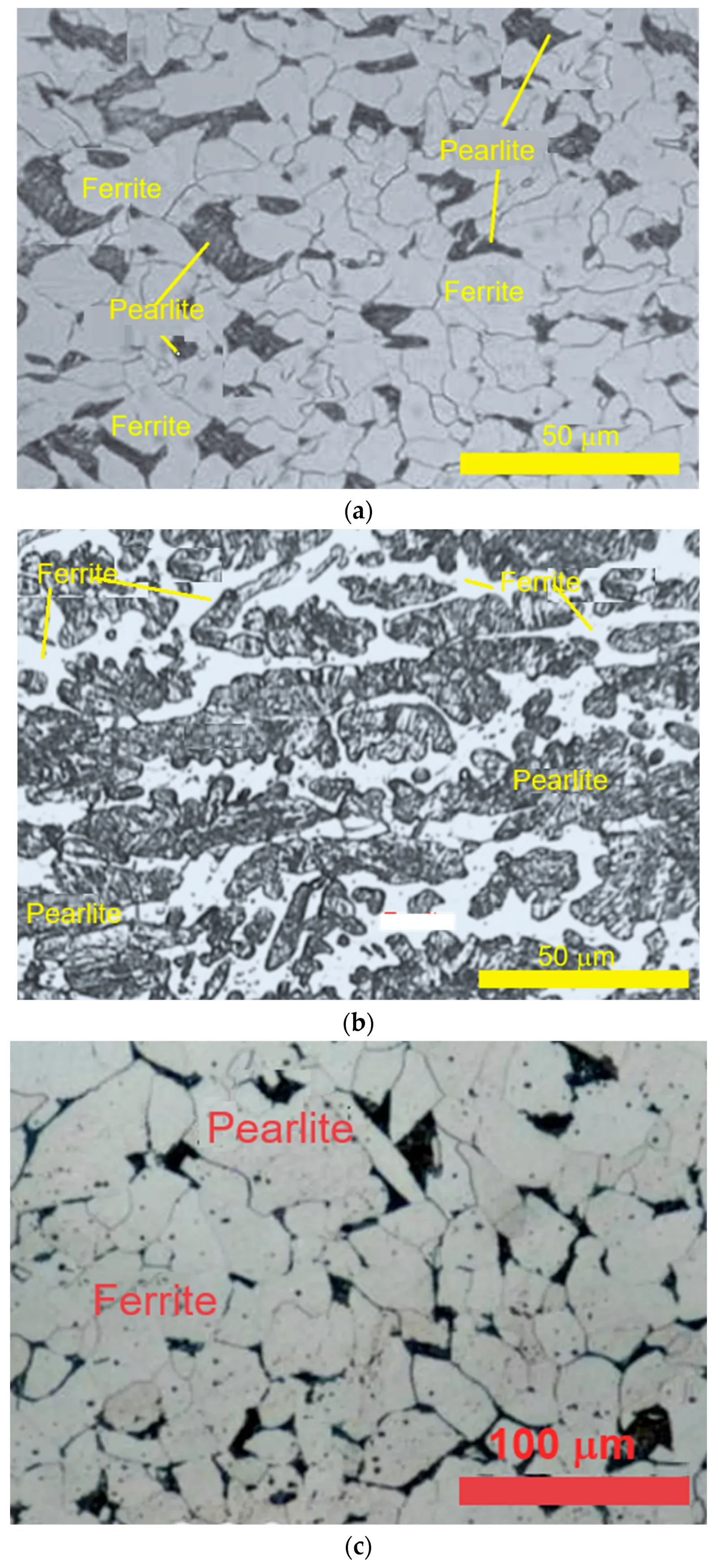
Microstructural images of the surface structures before cavitation exposure: (**a**) S235J2 general-purpose structural carbon steel. (**b**) GS300 cast steel. (**c**) C15E case-hardening quality carbon steel.

Figure 1 presents representative images of the microstructures observed on the surfaces of the cavitation specimens prior to experimental testing. The images were acquired using an OLYMPUS SZX57 stereomicroscope (Olympus Corporation, Hamburg, Germany) equipped with QuickPHOTO MICRO 2.2 image-processing software (Olympus Corporation, Hamburg, Germany) in the Materials Science Laboratory at Politehnica University of Timisoara (Timișoara, Romania).

The microstructural images presented in Figure 1 show that all three steels consist predominantly of ferrite (soft and ductile phase and is generally regarded as a constituent with low resistance to cavitation loading [27,28,42,46] and pearlite (consisting of a lamellar mixture of ferrite and cementite and exhibiting higher mechanical strength [47,48,49]). Using the lever rule, we estimate that among the investigated steels, GS300 cast steel contains the highest proportion of pearlite (the dark color-gray), approximately 80%, whereas C15E quality carbon steel exhibits the highest ferrite content (the light-colored constituent-white), approximately 85%.

Stereomicroscopic analysis and measurements reveal distinct microstructural characteristics for the three steels. The S235J2 steel, supplied as a rolled semi-finished product, exhibits an oriented grain structure with in-plane dimensions ranging from approximately 5–25 μm, with pearlite distributed as discrete islands. The GS300 cast steel exhibits a coarse-grained structure characteristic of the casting process used to produce the semi-finished material, with in-plane grain dimensions exceeding 150 μm, in some regions. In the normalized C15E quality carbon steel, supplied as a rolled semi-finished product, the ferrite grains reach dimensions of up to approximately 80 μm, whereas the pearlite regions, distributed as islands within the ferritic matrix, reach dimensions of up to approximately 40 μm. Based on these observations, and as demonstrated by earlier studies by Edel et al. [2], Hammitt [20], and Kornfeld & Suvorov [19], as well as more recent investigations by Bordeasu et al. [42,46,50,51], Cuppari et al. [52], and Marc et al. [21], the extent of material degradation caused by the cavitation erosion mechanism is strongly influenced by several material-related factors. These include the microstructure, particularly its phase constitution and grain size; the content of alloying elements such as Cr, Ni, and Cu; the carbon content; and the mechanical properties, particularly surface hardness.

## 3. Experimental Research

For S235J2 steel, the present investigation provides additional insights beyond those reported by Krella et al. [28] for S235JR steel, which has comparable mechanical properties and chemical composition. This is particularly relevant because the present experiments were conducted using a vibratory cavitation apparatus, which generates substantially higher cavitation erosion intensity than the rotating-disc device and hydrodynamic tunnel used by Krella.

### 3.1. Research Infrastructure and Methodology

**Figure 2:**
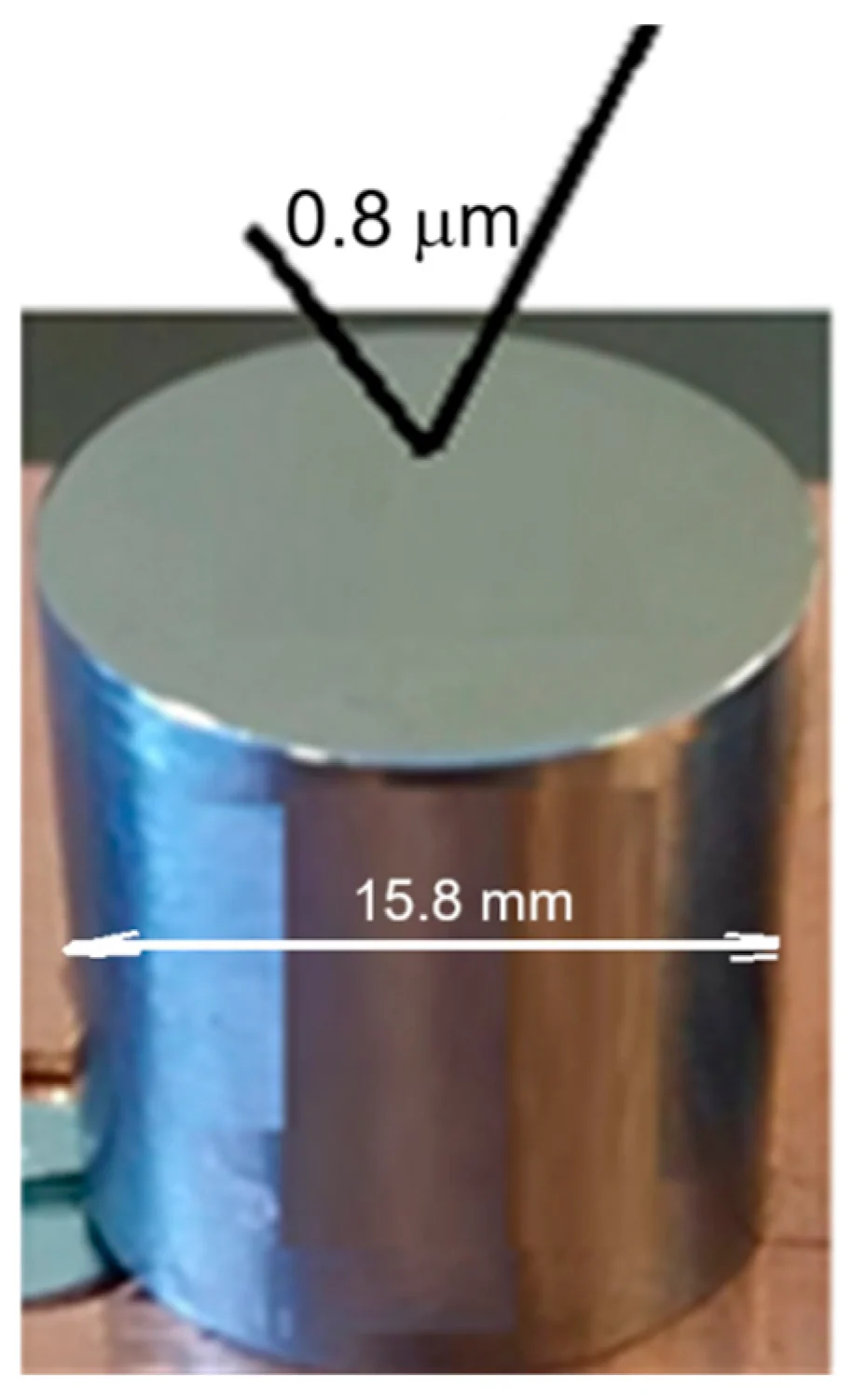
Image of the specimen surface before the cavitation exposure (Reprinted from Ref. [50]).

The cavitation erosion tests were conducted at the Cavitation Erosion Research Laboratory of Politehnica University Timișoara, Romania, using a standard vibratory cavitation device equipped with piezoceramic crystals. The main operating parameters were as follows: ultrasonic electronic generator power of 500 W, vibration amplitude of 50 μm, vibration frequency of 20 ± 0.02 kHz, and distilled-water temperature of 22 ± 1 °C) [9]. The experiments were performed using the direct method (with vibrating specimen) in accordance with the requirements of ASTM G32-2016 [53], the international standard for vibratory cavitation erosion testing. The experimental procedure followed the established testing protocol of the laboratory [9,54]: Three specimens of each steel were tested for a total cavitation exposure time of 165 min. The total exposure time was divided into 12 testing intervals: the first two intervals were 5 and 10 min, respectively, followed by ten intervals of 15 min each. Cylindrical specimens with a diameter of 15.8 mm were used (Figure 2). After each testing interval, the mass loss due to cavitation erosion was determined by weighing the specimens using a Kern ABT100-5NM analytical balance (KERN & SOHN GmbH, Balingen, Germany) with a resolution of 10^−5^ g. The operating parameters of the vibratory apparatus were monitored using dedicated software, while the experimental data were recorded and processed using Mathcad 15.

Note. The dedicated software, developed by Bordeasu and its team [9,34,55], is implemented on the computer connected to the vibratory cavitation apparatus. The software maintains the main operating parameters (the electrical power supplied to the piezoceramic transducer, vibration amplitude and frequency, and distilled-water temperature) at their prescribed values (mentioned above).

To investigate the evolution of surface degradation during the intermediate testing periods and after completion of the cavitation test, both macroscopic and microscopic images were acquired. The macroscopic images were obtained using a high-resolution Canon Power Shot A480 digital camera, while the microscopic images were acquired using the scanning electron microscope (SEM) TESCAN VEGA 3 LMU Bruker EDX Quantax (Tescan, Brno, Czech Republic).

### 3.2. Experimental Results and Discussion

#### 3.2.1. Morphology of the Cavitation-Eroded Structure

**Figure 3:**
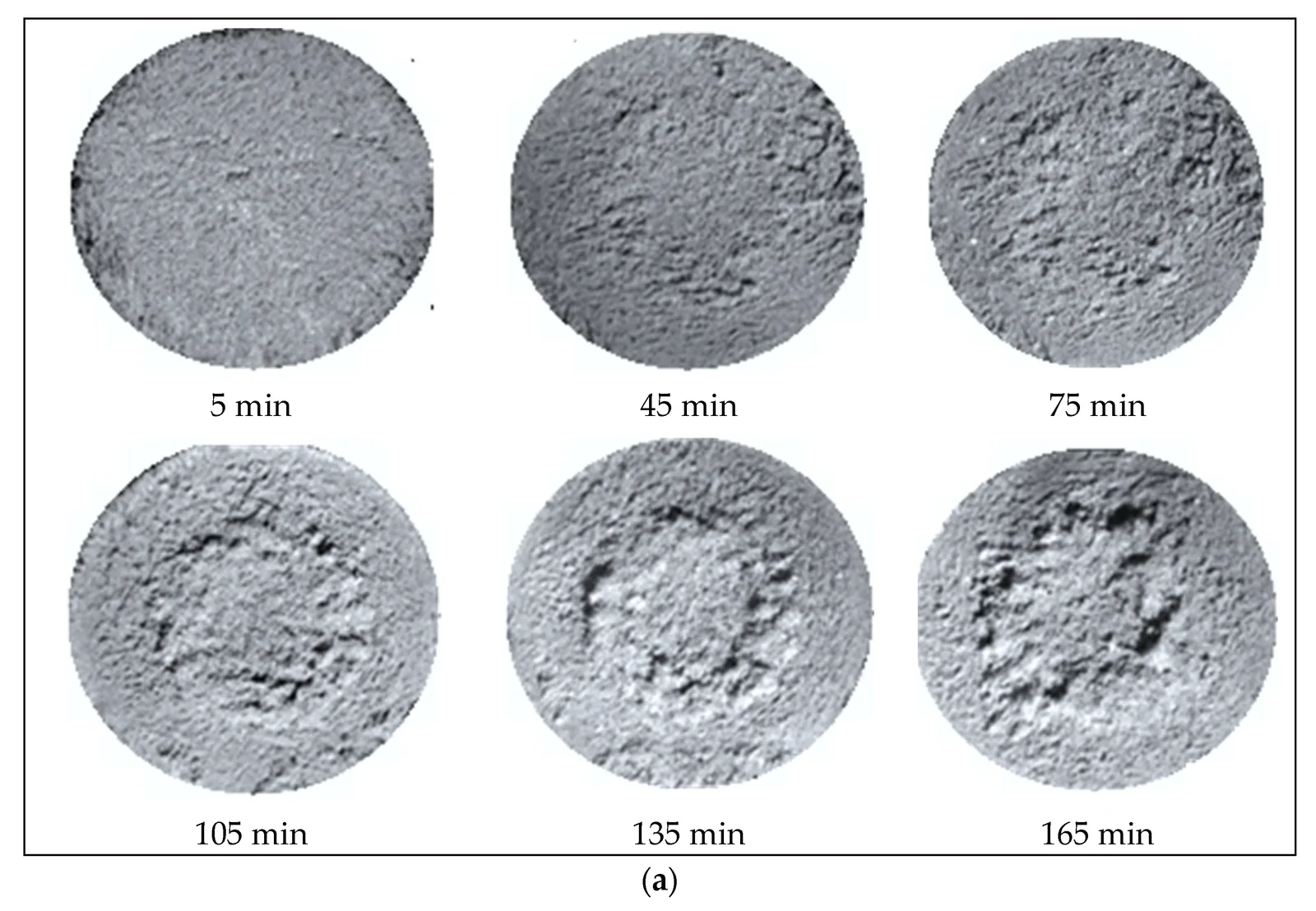

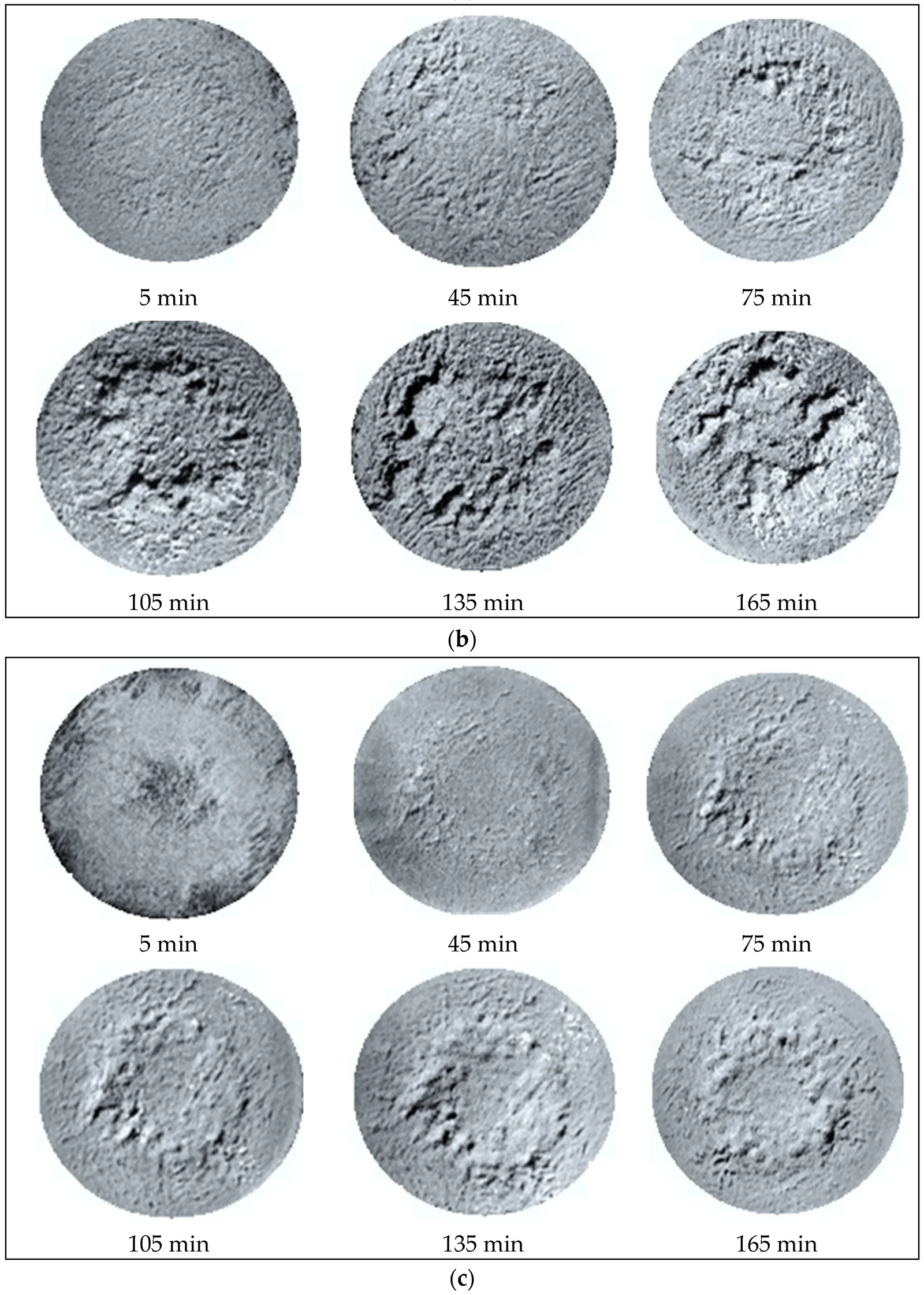
Evolution of cavitation erosion on the exposed surface (photos taken using a Canon Power Shot A480 camera—Canon Inc. Tokyo, Japan) of (**a**) S235J2 general-purpose structural carbon steel; (**b**) GS300 cast steel; (**c**) C15E case-hardening quality carbon steel.

**Figure 4:**
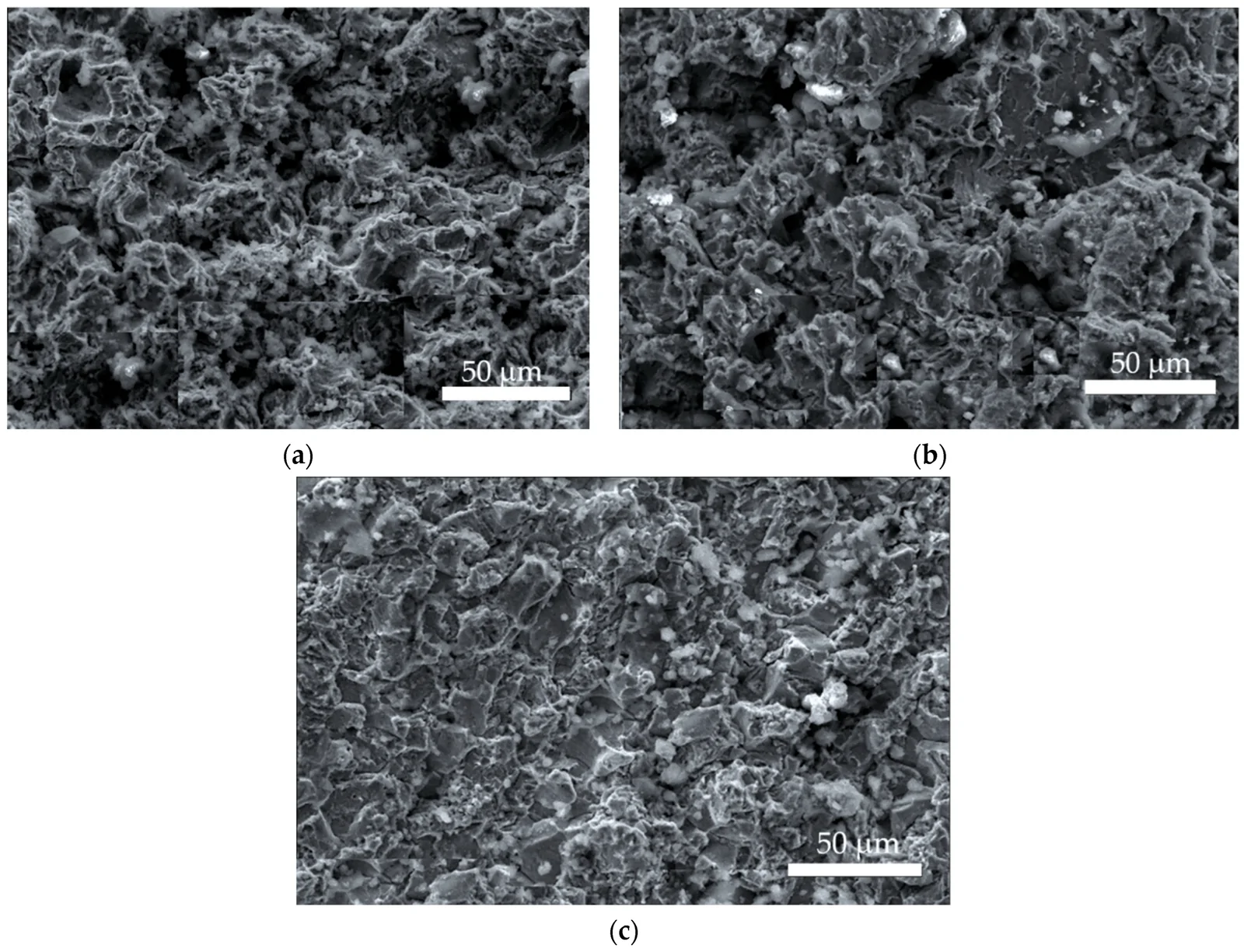
SEM images (×100 zoom) of cavities formed on the eroded surface after 165 min of cavitation exposure: (**a**) S235J2 general-purpose structural carbon steel. (**b**) GS300 cast steel. (**c**) C15E case-hardening quality carbon steel.

**Figure 5:**
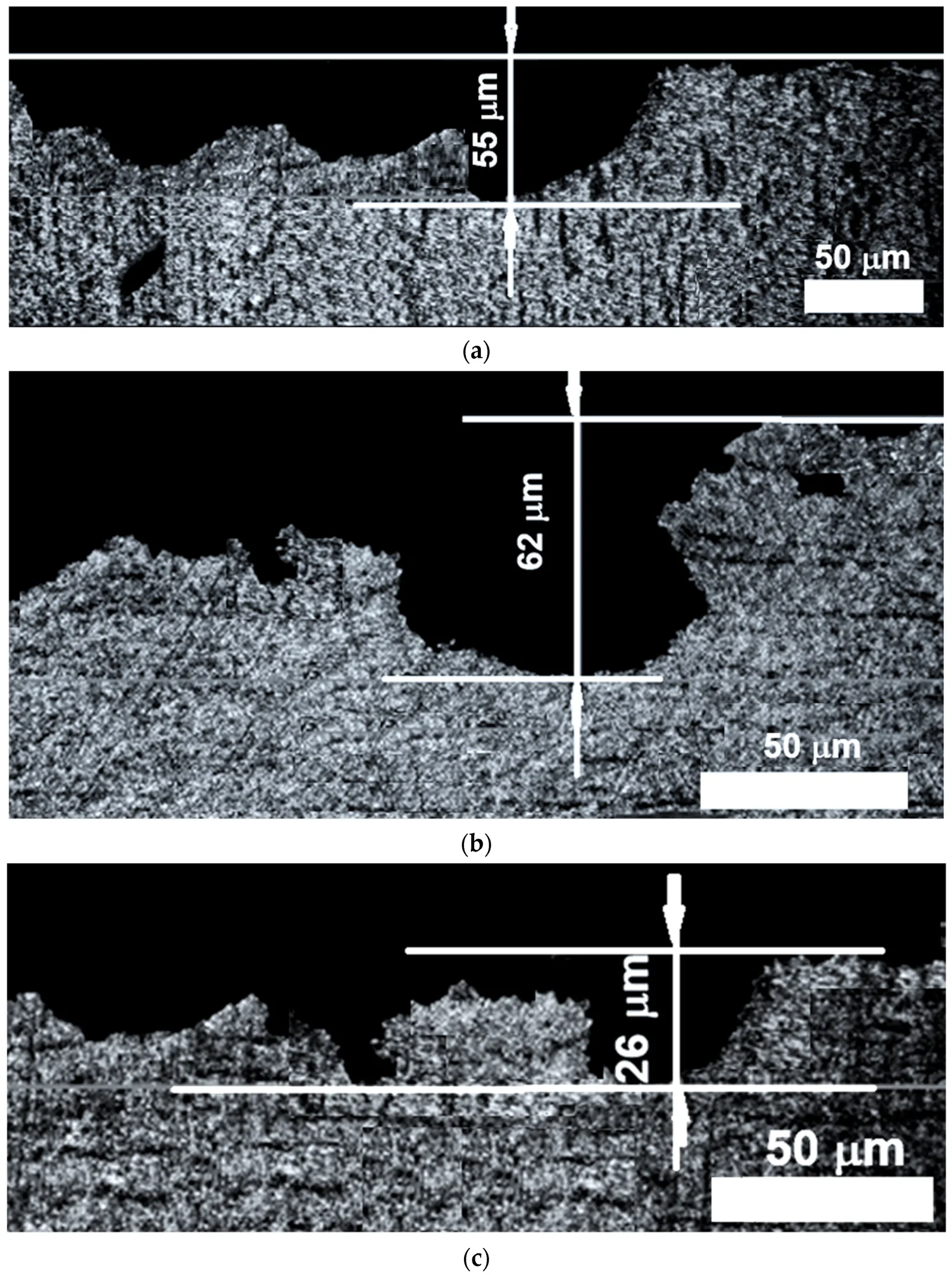
CT images illustrating cavity depth distribution of (**a**) S235J2 general-purpose structural carbon steel; (**b**) GS300 cast steel; (**c**) C15E case-hardening quality carbon steel.

The morphology of the cavitation-eroded surfaces was investigated using the macroscopic images presented in Figure 3 by the scanning electron microscopy (SEM) images shown in Figure 4 and by X-ray computed tomography (CT) shown in Figure 5. The macroscopic images reveal the progressive degradation of the surface structure with increasing cavitation exposure time, resulting from the repeated action of shock waves and microjets generated by the implosion of cavitation bubbles. The SEM images provide more detailed information on the mechanisms governing cavity formation through localized deformation, cracking, and material removal within the ferritic and pearlitic constituents.

Macroscopic images were acquired immediately after completion of each intermediate cavitation exposure interval at the Cavitation Erosion Research Laboratory.

**Figure 6:**
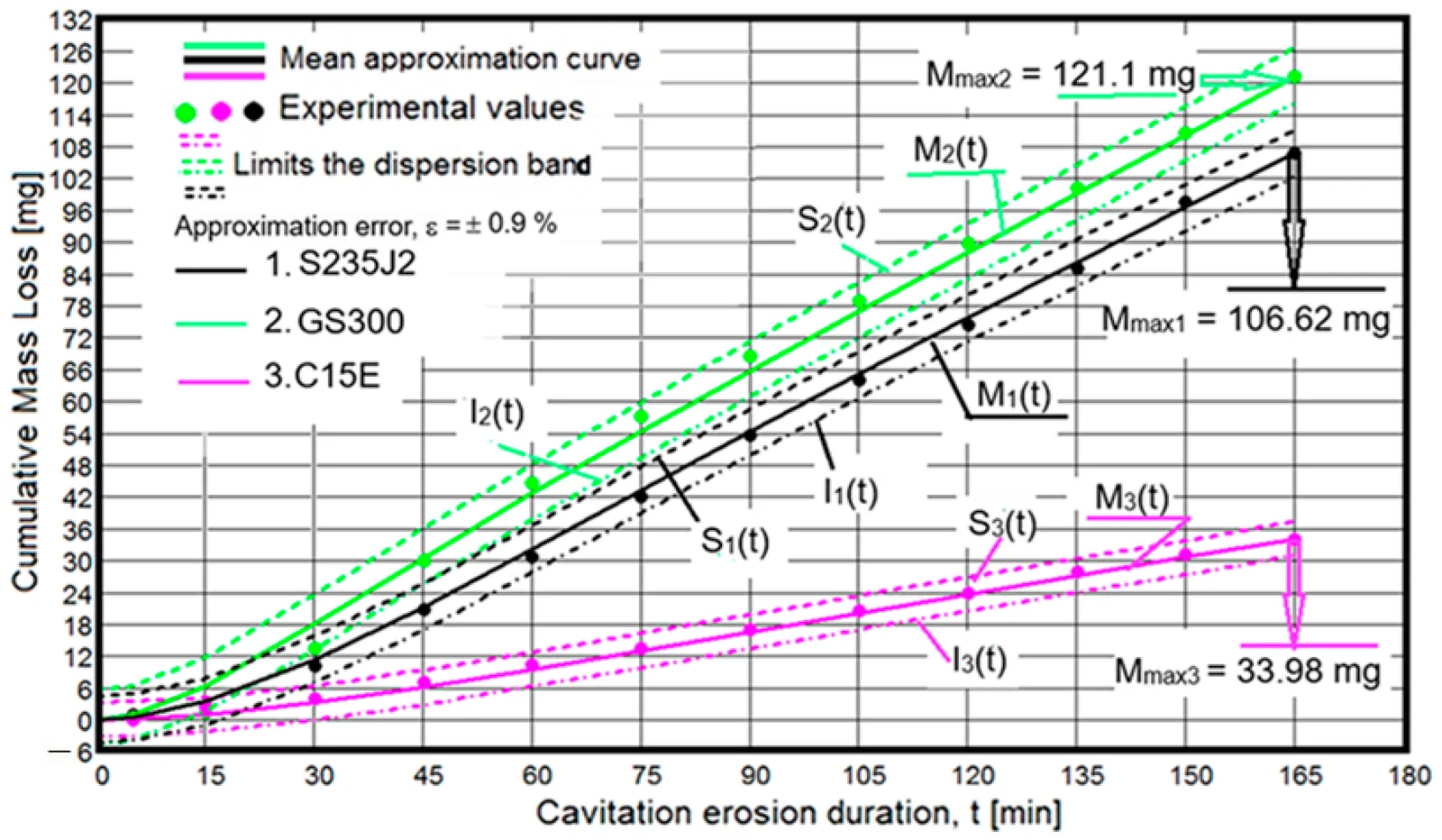
Variation in cumulative mass loss with cavitation exposure time.

**Figure 7:**
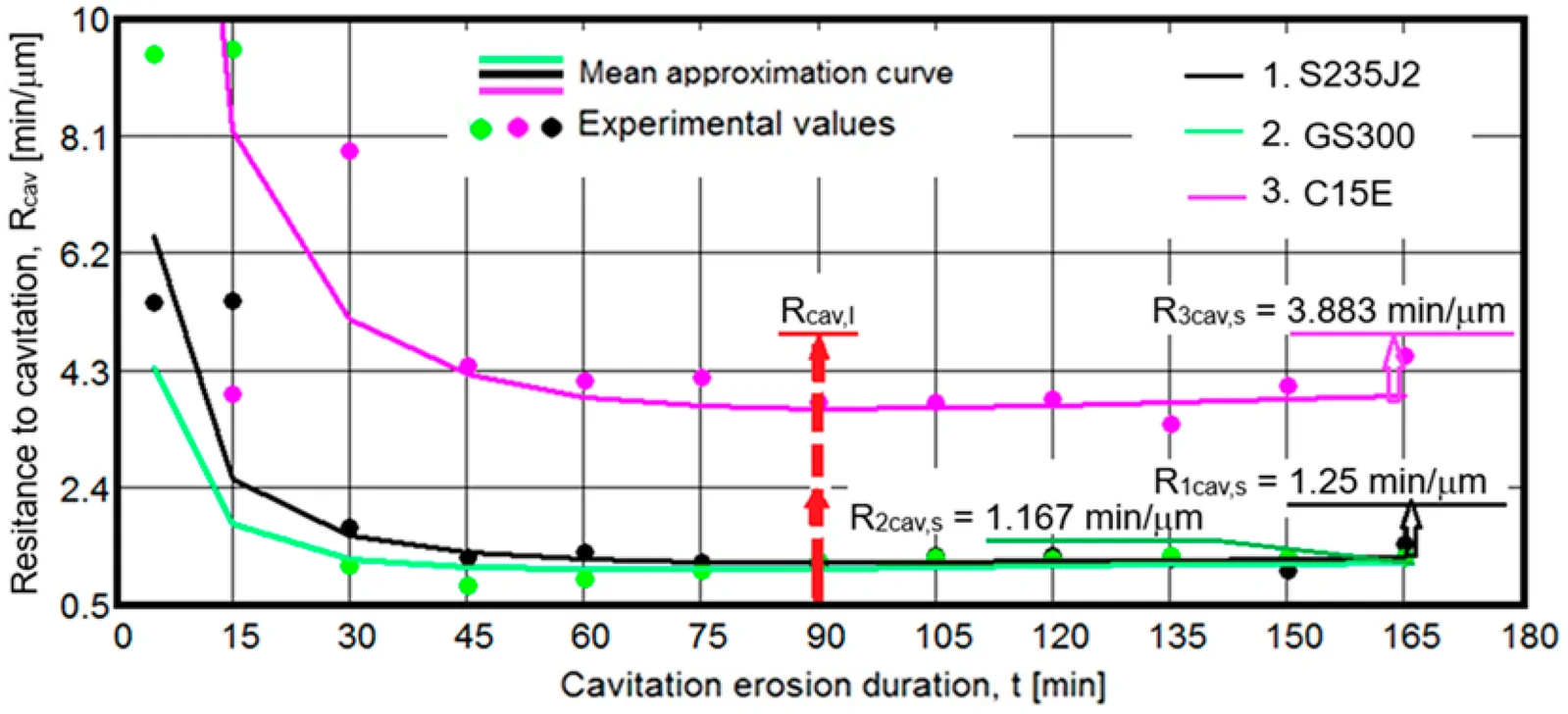
Variation in cavitation erosion resistance with cavitation exposure time.

Of the 12 images acquired during the test, 6 representative images were selected to illustrate the evolution of surface degradation, using the initial surface condition shown in Figure 2 as a reference. When correlated with the characteristic cavitation erosion curves presented in Figure 6 and Figure 7, these images provide insight into the response and resistance of the material microstructure throughout the cavitation erosion process.

The SEM images presented in Figure 4 were acquired using a TESCAN VEGA 3 LMU Bruker EDX Quantax scanning electron microscope (Tescan, Brno, Czech Republic). The observations were performed within the cavitation-eroded area after completion of the total exposure time of 165 min. Their examination supported the fractographic analysis and provided information on the response of the two microstructural constituents, ferrite and pearlite, to the impact pressures, as well as on the development of deformations and cracks leading to bond breaking, grain detachment and material removal, and ultimately to the formation and progressive enlargement of erosion cavities.

The depth of the erosion cavities (Figure 5) was investigated by X-ray computed tomography (CT) using a TESCAN Unitom HR scanner (Tescan, Brno, Czech Republic) equipped with Panthera 1.4.4. specialized image-processing software.

The images presented in Figure 3 show that the cavitation-eroded surfaces exhibit an irregular topography, with the larger erosion cavities predominantly concentrated in the central region of the exposed surface. This damage distribution is attributed to the characteristic shape and spatial distribution of the cavitation bubble cloud attached to the specimen surface [44,56]. Based on the progressive development of the eroded area and the size of the erosion cavities, C15E exhibits higher cavitation erosion resistance compared with the other two steels, despite its higher ferrite content. This behavior is further confirmed by the differences observed among the M(t) and R_cav_(t) curves and by the corresponding values of the characteristic parameters M_max_ and R_cav,s_ presented in Figure 6 and Figure 7.

According to the findings reported by Hobbs [57], Steller [58] and Bordeasu [45], the superior cavitation erosion resistance of the rolled C15E steel can be associated with its higher hardness and lower elongation at fracture. As shown in Table 2, its hardness is approximately 8–27% higher, while its elongation at fracture is 10% lower than that of GS300 steel and 33.3% lower than that of S235J2 steel. The beneficial influence of increased hardness is also supported by the findings of Krella [28], who comparatively investigated S235JR steel in its semi-finished state, with a hardness of 148.5 HV_0.5_, and the same steel after bulk heat treatment at 930 °C, which resulted in a lower hardness of 106 HV_05_.

Furthermore, compared with the rolled S235J2 general-purpose structural steel, the images presented in Figure 3 reveal more severe cavitation degradation of the GS300 cast steel, despite its higher pearlite content, which is associated with an approximately 18% higher hardness and a 35% lower elongation at fracture. This more pronounced degradation is attributed to the chemical composition and larger grain size resulting from the manufacturing process of the semi-finished material.

Accordingly, the lower cavitation erosion resistance of GS300 cast steel can be associated with the combined influence of several factors: its higher carbon content (0.27%, Table 1), an element that promotes microstructural embrittlement and weakens intergranular bonding [30,38]; the presence and relative contents of Ni, Cu, and Cr, which can influence microstructural strengthening and intergranular cohesion [47,59]; and the considerably larger grain size resulting from the casting process [2,47,59].

It should be noted that, in all cases, after 105 min of cavitation exposure, the differences in the size of the erosion cavities and the eroded area become relatively small. This behavior can be attributed to the work-hardening of the exposed surface, as also evidenced by the slight increase in the R_cav_(t) curves over the R_cav,i_–R_cav,s_ interval (Figure 7). Another contributing factor is the attenuation of the impact pressures due to the air and water that penetrate and become trapped within the erosion cavities. Under repeated compression, the entrapped fluid contributes to the initiation and propagation of cracks that precede the rupture of intergranular and transgranular bonds [45,52,60,61,62].

The analysis presented above highlights the complexity of the mechanical response of steel microstructures to cyclic fatigue loading induced by cavitation [63] loading and demonstrates that cavitation erosion resistance is governed by the combined influence of mechanical properties, microstructural constitution and carbon content and the concentrations of alloying elements, as also observed by Tzanakis et al. [64] in their investigation of commercial steel grades. These findings provide a clear rationale for the application of both bulk and surface heat treatments, as well as advanced surface-engineering technologies, including coating deposition and laser- and TIG-based surface hardening. Such treatments modify the microstructure and mechanical properties of the surface layers exposed to cavitation erosion.

Scanning electron microscopy (SEM) analysis (check Figure 4) reveals a heterogeneous distribution of erosion cavities of various sizes, ranging from small to large cavities, resulting from the intergranular and transgranular propagation of cracks. The SEM images also reveal non-metallic inclusions, severe plastic deformation, microcracks along grain boundaries, and cleavage regions associated with transgranular fracture, particularly in the GS300 and S235J2 steels. These degradation features produce pronounced raised rims surrounding erosion cavities of various shapes.

These characteristic features of the cavitation-eroded surfaces are primarily associated with ferrite, a highly ductile microstructural constituent [28,38,47,48] with relatively low resistance to the extremely high local pressures, on the order of tens of GPa, generated by cavitation microjets and shock waves [25,43,62].

Microscopic examination of the deformation features, crack paths, and erosion-cavity geometry shown in Figure 4 supports the degradation mechanisms reported in previous studies [28,45,62,63,64,65,66,67,68]. According to these mechanisms, cracks preferentially initiate and propagate along ferrite–ferrite and ferrite–pearlite boundaries, while the formation of larger erosion cavities is associated with the detachment and removal of groups of grains or microstructural regions consisting of ferrite or ferrite and pearlite. This interpretation is consistent with the intrinsic characteristics of the constituent phases and with the mechanical properties of the investigated steels reported in Table 2.

The pronounced degradation of the cavitation-eroded surface of S235J2 steel, evidenced by the large erosion cavities also visible in the SEM image (Figure 4a), is characteristic of microstructures exhibiting relatively low cavitation erosion resistance [20,38]. According to the literature [21,28,38,48], this behavior can be attributed to relatively weak intergranular cohesion and the severe plastic deformation that develops under repeated cavitation loading. These degradation mechanisms are promoted by the comparatively low values of tensile strength (R_m_), yield strength (R_p02_), and hardness (HB), together with the relatively high elongation at fracture (A_5_).

The relatively low cavitation erosion resistance of GS300 steel is particularly noteworthy because this material exhibits the highest tensile strength (R_m_) and yield strength (R_p02_) among the three investigated steels, as well as higher hardness and lower elongation at fracture than S235J2 steel (check Table 2). As discussed previously, this behavior is attributed primarily to its relatively high carbon content (0.27%, check Table 1), which promotes microstructural embrittlement [2,47,59], and to its coarse-grained microstructure, characterized by the large grain sizes typically associated with cast materials [2].

Another factor contributing to the lower cavitation erosion resistance of S235J2 and GS300 steels is the presence of non-metallic inclusions (visible as white regions in the SEM images) which weaken the bonds between grains [44,45].

The SEM fractographic analysis of C15E steel reveals a relatively uniform erosion morphology, characterized by erosion cavities markedly smaller than those observed in S235J2 and GS300 steels, together with features associated with both brittle and ductile fracture. Its superior cavitation erosion resistance is attributed primarily to its higher hardness, which has been identified as one of the mechanical properties exerting the strongest positive influence on the resistance of surfaces exposed to cavitation, and which reaches the highest value among the three investigated steels (check Table 2). A further contributing factor is its lower elongation at fracture, which exhibits the lowest value among the investigated materials (check Table 2), a parameter that contributes to increasing cavitation resistance when its value is as low as possible [21,45,68].

The measurements were performed using a TESCAN VEGA 3 LMU Bruker EDX Quantax scanning electron microscope (Tescan, Brno, Czech Republic). As illustrated in Figure 5, representative erosion cavities with the smallest and largest in-plane dimensions were selected at arbitrary locations within the eroded area and measured by SEM. The cavity depths were subsequently determined by X-ray computed tomography (CT) using a TESCAN UniTOMHR (Tescan, Brno, Czech Republic). The measurements revealed maximum cavity dimensions of up to 175 μm in length and 58 μm in depth for S235J2 general-purpose structural steel; up to 210 μm in length and 62 μm in depth for GS300 cast steel; and up to 35 μm in length and 30 μm in depth for C15E steel.

It should be noted that, regardless of the steel type, some erosion cavities exhibit tunnel- or cave-like morphologies, extending either perpendicular or inclined to the cavitation-eroded surface. Identifying the specific microstructural constituent responsible for the formation of this type of cavity is difficult because of the spatial distribution and intermixing of the two principal phases, ferrite and pearlite, within the eroded surface microstructure. According to previous studies [50,51,69] these characteristic morphologies can be attributed to an explosion-like effect produced by water and air trapped within previously formed erosion cavities, compressed by the high pressures exerted by the cavitation microjets.

In conclusion, the similarities observed among the cavitation erosion morphologies of the investigated steels are primarily governed by their microstructural constitution, whereas the differences can be attributed to variations in hardness, elongation at fracture, the carbon content and the concentrations of alloying elements.

#### 3.2.2. Cavitation Erosion Characteristic Curves

The characteristic curves used to evaluate the cavitation erosion behavior and resistance of the investigated steels are presented in Figure 6 and Figure 7. In accordance with the requirements of ASTM G32-2016 [53] and the established testing and data-processing methodology of the laboratory, these diagrams include the experimental values, calculated as the arithmetic means of the measurements obtained from the three specimens tested for each steel, together with the fitted M(t) and R_cav_(t) curves. They also provide the characteristic parameters M_max_ and R_cav,s,_ which are used to quantify and compare the resistance of the investigated steels to the loading generated by the microjets and shock waves resulting from cavitation bubble implosion.

Figure 6 presents the cumulative mass loss recorded over the total cavitation exposure time of 165 min. To demonstrate the accuracy of the experimental results and the quality of their mathematical representation, the diagram also includes the approximation error (ε), which quantifies the deviation of the experimental data from the fitted M(t) curves. In addition, the S(t) and I(t) curves are plotted to define the dispersion bounds of the experimental data. These curves were constructed using the relationships developed at the Cavitation Erosion Research Laboratory [9,55,59,70]. The corresponding relationships are expressed as follows:

- For the variation in mass loss with cavitation exposure time,

M(t) = A⋅t⋅(1 − e^−B·t^), expressed in mg (1)

- For the standard deviation,

(2) $$\sigma ={\left[\frac{\sum_{i=1}^{n}{\left(M_{i}-M(t{)}_{i}\right)}^{2}}{n-1}\right]}^{\frac{1}{2}},\;\mathrm{expressed}\;\mathrm{in}\;\mathrm{mg}$$

The experimental values of cumulative mass loss, (M_i_), are determined algebraically from the mass losses measured using the analytical balance at the end of each intermediate cavitation exposure interval, according to the following relationship:

(3) $$\mathrm{Mi}=\Sigma_{i=1}^{n}\Delta m_{i},\;\mathrm{expressed}\;\mathrm{in}\;\mathrm{mg}$$

where Δm_i_ is the arithmetic mean of the mass losses recorded for the three specimens tested during the “i”-th exposure interval, expressed in mg.

M(t)_I_ represents the mass value corresponding to the point on the fitted curve at the exposure time associated with the “i”-th testing interval.

- For the curves defining the dispersion range of the experimental values,

S(t) = [M(t)] + ε⋅σ, expressed in mg I(t) = [M(t)] − ε⋅σ, expressed in mg(4)

where ε = 0.9 is a parameter determined iteratively to minimize the width of the dispersion band while ensuring that all experimental values fall within its boundaries. For this reason, the parameter ε is also referred to as the approximation error [45,49].

Figure 7 represents the variation in cavitation erosion resistance with cavitation exposure time, whose analytical expression is [51]:

(5) $$R_{\mathrm{cav}}(t)=\frac{1}{C\cdot \left(1-e^{-D\cdot t}\right)+C\cdot D\cdot t\cdot e^{-D\cdot t}},\;\mathrm{expressed}\;\mathrm{in}\;\min/\mu m$$

The experimental values of cavitation erosion resistance, R_cav i_, are determined based on the diameter of the surface exposed to cavitation (15.8 mm; see Figure 1), the mass-loss rate Δm_i_, calculated as the arithmetic mean for the three tested specimens during the “i”-th intermediate testing interval, and the duration of that interval, Δt_i_, according to the following equation:

(6) $$R_{{\mathrm{cav}}_{i}}=\frac{\rho \cdot \pi \cdot d_{p}^{2}\cdot {\Delta t}_{i}}{4\cdot {\Delta m}_{i}},\;\mathrm{expressed}\;\mathrm{in}\;\min/\mu m$$

Here, “i” denotes the index of the intermediate testing interval; ρ = 7810 kg/m^3^ is the steel density; dp = 12 mm is the diameter of the surface eroded; Δt_i_ is the duration of the “i”-th testing interval, expressed in minutes; and Δm_i_ represents the arithmetic mean of the mass losses recorded for the three specimens during the “i”-th testing interval, expressed in mg.

Table 3 presents the values of the coefficients used in Equations (1) and (5), together with the standard deviation, σ defined by Equation (2).

The approximation error (ε = ±0.9%) confirms the high reproducibility of the cavitation test under rigorously controlled experimental conditions, particularly with respect to the operating parameters of the vibratory apparatus, which govern the hydrodynamic intensity of cavitation, as well as the water temperature.

The data presented in Figure 6 and Figure 7 reveal both similarities and differences in the cavitation erosion behavior of the three investigated steels. These features are governed by their microstructural characteristics, mechanical properties, and the manufacturing route of the semi-finished material, namely rolling or casting.

The similarities are reflected in the dispersion of the experimental data around the fitted curves and in the characteristic shapes of the M(t) and R_cav_(t) curves. According to the findings reported in [21,62], the dispersion of the experimental values is closely related to the microstructural constitution of the materials, particularly the distribution of ferrite and pearlite. The M(t) curves tend toward an approximately linear evolution after about 30 min of cavitation exposure (Figure 6), whereas the R_cav_(t) curves exhibit a tendency toward stabilization after approximately 90 min (Figure 7). Based on the extensive experimental investigations conducted in our laboratory over more than 80 years [9], as well as findings reported in the literature [12,20,21,62], the evolution of these curves is governed by the resistance of the surface microstructure to cavitation, the mechanisms of microstructural degradation discussed in Section 3.2.1, and the progressive work-hardening of the surface layer under the repeated impact of cavitation shock waves and microjets. The tendency toward a nearly linear evolution of these curves, when correlated with the morphology of the eroded surfaces after 105, 135, and 165 min of cavitation exposure (Figure 3), can also be associated with the presence of air and water trapped within the erosion cavities. According to previous studies [45,51,62,69] the entrapped fluid is repeatedly compressed by the high local pressures generated by shock-wave and microjet impacts. This process partially attenuates the transmitted impact pressure while simultaneously producing an explosion-like effect within pre-existing cavities, which promotes localized deformation, crack initiation and propagation, and the subsequent detachment of grain fragments. Evidence supporting this mechanism is provided by the excavated morphology of the erosion cavities observed in the microscopic images (4 and 5).

As shown in Table 4, C15E steel, which exhibits the highest cavitation erosion resistance, undergoes the smallest increase in resistance during the test, indicating a lower degree of mechanically induced surface work-hardening. Conversely, GS300 steel, which exhibits the poorest cavitation erosion behavior, shows the greatest increase in resistance, suggesting a more pronounced work-hardening effect. This behavior can be related to differences in the ability of the investigated steels to undergo plastic deformation under repeated mechanical loading [53,54,71].

The differences in cavitation erosion behavior, and consequently in resistance to vibratory cavitation loading, are also reflected in the slopes of the M(t) and R_cav_(t) curves within their approximately linear regions, as well as in the differences between the characteristic comparison parameters M_max_ and R_cav,s_.

It is particularly noteworthy that S235J2 general-purpose structural steel and GS300 cast steel exhibit only minor differences in cavitation erosion behavior (as indicated by the mass-loss curves in Figure 6), while their behavior becomes approximately identical after 90 min of cavitation exposure, as shown in Figure 7. This observation highlights the complexity of cavitation erosion mechanisms, since the material response is governed by the simultaneous influence and interaction of numerous material-related factors, particularly the constituent microstructural phases and the physical and mechanical properties of the material. This interpretation is consistent with findings widely reported in the specialized literature and may also explain observations from cavitation erosion studies conducted in our laboratory, in which materials belonging to different classes, particularly metallic materials, have exhibited similar cavitation erosion behavior.

Based on the reference parameters recommended by ASTM G32-2016 and routinely used in our laboratory for evaluating cavitation erosion resistance, C15E steel exhibits a markedly higher resistance than the other two investigated steels. Based on the M_max_ values, its cavitation erosion resistance is approximately 3.56 times higher than that of GS300 cast steel and 3.13 times higher than that of S235J2 steel. Similarly, based on the R_cav,s_ values, the resistance of C15E steel is approximately 3.33 times higher than that of GS300 and 3.11 times higher than that of S235J2. The same reference parameters indicate that S235J2 carbon steel exhibits slightly higher cavitation erosion resistance than GS300 cast steel, by approximately 13.5% according to M_max_ and approximately 7% according to R_cav,s_. These differences are supported by the results presented in Section 3.2.1, particularly by the dimensions of the erosion cavities and the fractographic features of the cavitation damage.

## 4. Conclusions

The similarities and differences in cavitation erosion behavior, as well as the different levels of resistance exhibited by the three investigated carbon steels, S235J2, GS300, and C15E, under the destructive action of microjets and shock waves generated during vibratory cavitation are governed by the combined influence of their microstructural and mechanical characteristics. The most important factors include the relative proportions of ferrite and pearlite, mechanical properties, particularly surface hardness and elongation at fracture, the carbon content, the concentrations of alloying elements and grain size, especially that of the ferritic constituent.

Among the researched materials, C15E carbon steel exhibits the highest cavitation erosion resistance, exceeding that of S235J2 and GS300 steels by more than a factor of 3. This superior performance is associated with its chemical composition, more homogeneous and refined microstructure, higher hardness (HB = 150), and lower elongation at fracture (A5 = 18%).

The morphology and dimensions of the erosion cavities (Figure 3) reflect the relationship between cavitation erosion resistance and the ability of the material microstructure to absorb and dissipate the energy transferred by the impact of cavitation microjets and shock waves. The characteristic tunnel- and crater-like morphologies observed in Figure 4 and Figure 5 are also consistent with the proposed explosion-like mechanism associated with the rapid compression of air and/or water trapped within pre-existing erosion cavities under the action of cavitation microjets and shock waves.

Based on the morphological characteristics of the eroded surfaces, the extensive experimental experience accumulated in our laboratory, and findings reported in the literature, all three steels exhibit relatively low resistance to high-intensity cavitation erosion. Accordingly, their applications are generally associated with components subjected to less severe cavitation conditions. Typical examples include runner hubs and nose cones of hydraulic machines, spiral casings of turbopumps, and valve bodies manufactured from GS300 cast carbon steel; guide-vane rings, guide-vane bodies, and butterfly-valve discs manufactured from S235J2 carbon steel; and valve spools and seats of hydraulic control and regulating devices manufactured from C15E carbon steel. In order to extend the use to parts working in cavitational currents with higher intensity of erosion (moderate), it is necessary to continue the research on states resulting from the application of classical and new technologies that improve mechanical properties and microstructure. As surface hardness is indicated by the studies of Hammitt [20], Sarkar [62], Hobbs [57], Krella [28], Franc [21], etc., as the main property with a positive influence on cavitation strength, we consider that the study of its effect on these three categories of steels should be continued.

## Figures and Tables

**Table 1:** Chemical composition of the researched steels (values expressed in wt.%).

Steel Type	C	Si	Mn	Ni	S	P	Cr	Cu	Fe
S235J2	0.18	0.42	1.21	-	0.032	0.031	-	-	Rest
GS300	0.27	0.28	0.52	-	0.025	0.022	-	-	Rest
C15E	0.15	0.21	0.42	0.21	0.031	0.027	0.12	0.14	rest

**Table 2:** Mechanical properties of the researched steels.

Steel Type	R_m_ [MPa]	R_p0.2_ [MPa]	A_5_ [%]	HB [daN/mm^2^]
S235J2	367	215	27	118
GS300	490	280	20	139
C15E	470	265	18	150

**Table 3:** Values of the statistical coefficients used in Equations (1) and (5).

Steel Type	A	B	C	D	σ [mg]
S235J2	0.652	0.029	0.75	0.022	10.968
GS300	0.734	0.057	0.836	0.031	6.097
C15E	0.211	0.023	0.24	0.022	4.035

**Table 4:** Increase in surface resistance induced by mechanical cavitation loading.

Steel Type	S235J2	GS300	C15E
R_cav,i_	1.174	1.077	3.671
R_cav,s_	1.25	1.167	3.883
Increase in cavitation erosion resistance ΔR_cav_ = ((R_cav,s_ − R_cav,i_)⋅100/R_cav,i_), %	6.47	8.35	5.77

## Data Availability

The original contributions presented in this study are included in the article. Further inquiries can be directed to the corresponding authors.
